# Biosynthesis and signalling functions of central and peripheral nervous system neurosteroids in health and disease

**DOI:** 10.1042/EBC20200043

**Published:** 2020-08-05

**Authors:** Emyr Lloyd-Evans, Helen Waller-Evans

**Affiliations:** 1 Sir Martin Evans Building, School of Biosciences, Cardiff University, Museum Avenue, Cardiff, CF10 3AX, U.K.; 2 Medicines Discovery Institute, Main Building, Cardiff University, Park Place, Cardiff, CF10 3AT, U.K.

**Keywords:** Alzheimers disease, GABA, glutamate receptor, multiple sclerosis, Neurosteroid, Niemann Pick type C

## Abstract

Neurosteroids are steroid hormones synthesised *de novo* in the brain and peripheral nervous tissues. In contrast to adrenal steroid hormones that act on intracellular nuclear receptors, neurosteroids directly modulate plasma membrane ion channels and regulate intracellular signalling. This review provides an overview of the work that led to the discovery of neurosteroids, our current understanding of their intracellular biosynthetic machinery, and their roles in regulating the development and function of nervous tissue. Neurosteroids mediate signalling in the brain via multiple mechanisms. Here, we describe in detail their effects on GABA (inhibitory) and NMDA (excitatory) receptors, two signalling pathways of opposing function. Furthermore, emerging evidence points to altered neurosteroid function and signalling in neurological disease. This review focuses on neurodegenerative diseases associated with altered neurosteroid metabolism, mainly Niemann-Pick type C, multiple sclerosis and Alzheimer disease. Finally, we summarise the use of natural and synthetic neurosteroids as current and emerging therapeutics alongside their potential use as disease biomarkers.

## Introduction

**Figure 1:**
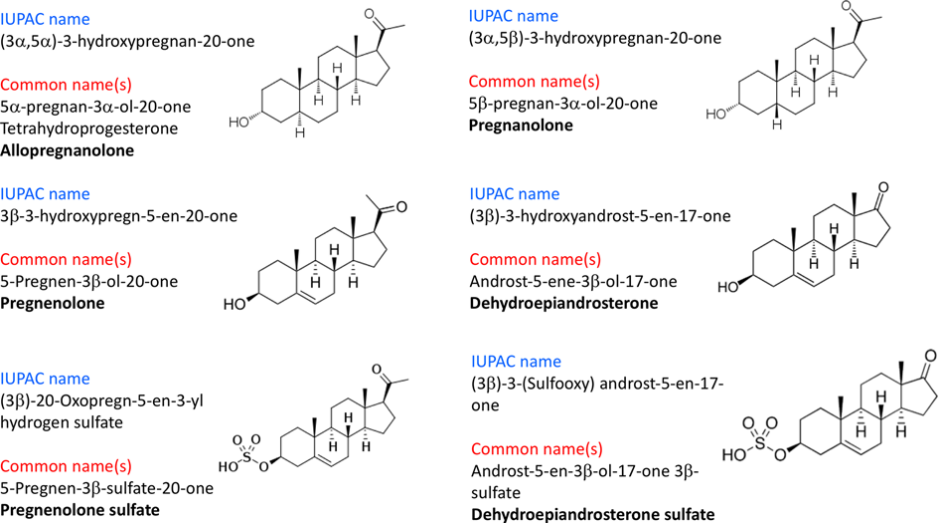
IUPAC and common nomenclature of certain pregnane, androstane and sulphated neurosteroids

It is now accepted, from knowledge accrued over the past 30–40 years, that the brain is a site of *de novo* steroid biosynthesis [1,2]. This means that the brain is a steroidogenic organ akin to the peripheral steroidogenic tissues, namely the adrenal glands, the gonads (ovaries and testes) and also the placenta [3]. The so called ‘neurosteroids’ are generated by largely the same mechanisms, but are produced specifically in the central nervous system (CNS) and the peripheral nervous system (PNS), either endogenously or from circulating sterols [4,5]. The term ‘neurosteroids’, coined in 1981 [4,6], does not refer to a different class of steroids but is instead a catch-all term used to describe androstane and pregnane-derived neurosteroids [7,8] and some of their sulphated forms [8] (Figure 1). Peripheral steroid hormones, such as cortisol, estrogen and testosterone are also neuroactive. However, these mostly act upon intracellular nuclear hormone receptors [9], with only limited evidence of peripheral steroid hormone interaction with plasma membrane proteins [10], to mediate subsequent changes in function of both peripheral and CNS tissues [11]. Neurosteroids are different to peripheral steroid hormones, as several have no identified nuclear hormone receptor (e.g. allopregnanolone) and they generally act directly upon plasma membrane ion channels and on mechanisms of neurotransmitter homeostasis to directly modulate neuronal excitability [12]. This review will focus specifically on CNS neurosteroids, their synthesis and biological roles; the mechanisms of action of steroids in peripheral tissues are not covered here as detailed recent reviews exist [13–15].

### The discovery of de novo brain ‘neurosteroid’ synthesis

The characterisation of the brain as a site of sterol synthesis was pioneered by the group of Baulieu. They identified that brain content of certain steroids and their sulfate esters, particularly dehydroepiandrosterone (DHEA), progesterone and to a lesser extent pregnenolone, did not drop in castrated rats that had also undergone adrenalectomy [4,6]. In contrast, the levels of testosterone and testosterone sulfate were quickly eliminated. When taken together with other findings that (1) the persistency of these brain sterols was unrelated to pools of circulating sterols, as seen by the rapid elimination of peripherally administered radioactive DHEA and pregnenolone in rats [16,17]; (2) the concentration of certain brain sterols is higher in the CNS than in peripheral fluids [17,18]; (3) brain pregnenolone levels are higher in newborn rats for several days despite low levels of sterol biosynthesis in the adrenal organs [4,16]; and (4) a circadian pattern of brain DHEA and pregnenolone exists separate to the periphery [19]. These data suggested that the brain could act as a *de novo* site of sterol synthesis. Subsequent work, detailed below, highlighted that neurosteroid biosynthesis was initially believed to be restricted to glia, which was later expanded to astrocytes, neurons of the hippocampus and cerebellum, and the pineal gland. Brain neurosteroid synthesis has been postulated to be necessary during times when circulating steroid hormone levels are low, e.g. occasionally during the female oestrous cycle, or to compensate for constitutively lower levels of progesterone in males [5]. However, neurosteroids are very lipophilic and are therefore less likely to be secreted into the circulation and more likely to be retained within the membranes of their synthetic cells, indicating that their actions are likely to be more local [20]. The ‘local’ biological requirement for neurosteroids in the brain is an emerging area, especially regarding signalling (discussed below). The importance of neurosteroid biosynthesis is further evidenced by the high degree of conservation of this process across many species including birds [21], amphibians [22] and fish [23]. This review will focus solely on their roles in mammals.

### The neurosteroid biosynthetic pathway

**Figure 2:**
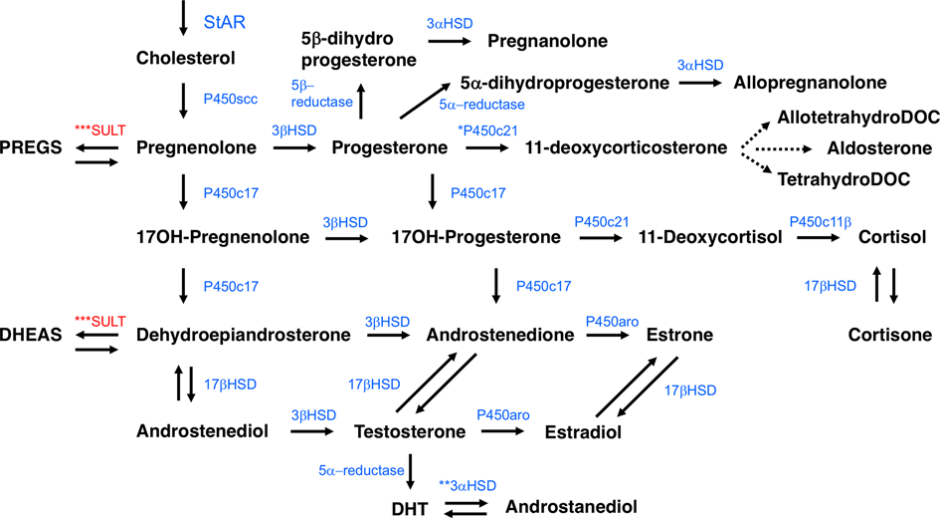
The central and peripheral nervous system biosynthetic pathway of neurosteroids The process is activated by transport of cholesterol into mitochondria by StAR and continues in the endoplasmic reticulum following production of progesterone. Biosynthetic enzymes are denoted in blue, neurosteroids in black and enzymes involved in sulphation in red. Note, synthesis *only reported in astrocytes, **only reported in Purkinje cells, ***only reported in microglia. Abbreviations: AllotetrahydroDOC, allotetrahydrodeoxycorticosterone; DHEAS, dehydroepiandrosterone sulphate; DHT, 5α-dihydrotestosterone; P450c11β, sterol 11β-hydroxylase; P450c21, steroid 21-hydroxylase; PREGS, pregnenolone sulphate; TetrahydroDOC, tetrahydrodeoxycorticosterone.

Steroidogenesis in neurons and glia of the CNS and PNS begins following transport of cholesterol, either sourced from *de novo* biosynthesis at the endoplasmic reticulum (ER) or from endocytosed low-density lipoproteins (LDL), into the mitochondria [24,25] (Figure 2). Alternatively, some sterols found in the circulation (e.g. progesterone, DHEA) can cross the blood–brain barrier (reviewed in detail here [26]) and are utilised as precursor molecules in the neurosteroid biosynthetic pathway [27]. The precise contribution of these precursors to neurosteroid synthesis remains to be fully examined [26]. Within the cell, mitochondria are the essential site of neurosteroidogenesis, which involves multiple cytochrome P450 enzymes (Figure 2) [28]. Cholesterol transport from the outer to inner mitochondrial membrane, mediated via the action of the steroidogenic acute regulatory protein (StAR), exerts a strong control over the neurosteroid biosynthetic pathway. StAR is anchored to the outer membrane of the mitochondria where it is complexed to the translocator protein (TSPO), also known as the peripheral benzodiazepine receptor [25]. Perhaps, the strongest evidence for the requirement of the StAR protein in steroid biosynthesis comes from studies of lipoid congenital adrenal hyperplasia patients, where mutations in the gene encoding StAR leads to a substantial defect in the conversion of cholesterol to pregnenolone [29]. The TSPO protein is localised, in a sterol hormone-dependent manner, to the outer mitochondrial membrane at the contact site with the inner mitochondrial membrane and is suggested, from reconstitution experiments, to act as a pore for cholesterol mobilisation to the inner mitochondrial membrane [30]. Furthermore, the structure of TSPO has been solved; it contains a cholesterol recognition/interaction amino acid consensus (CRAC) domain on the cytosolic C-terminus, and has been shown to bind cholesterol with high affinity [31,32]. However, TSPO knockout mice are viable, suggesting the possibility of redundant mechanisms for mitochondrial steroid hormone transport and biosynthesis [33]. Regardless of the mechanism that mediates cholesterol transport, it is here on the inner mitochondrial membrane that the first enzymatic step in steroid biosynthesis occurs. The cytochrome P450 side chain cleavage (P450scc) enzyme generates pregnenolone (a 3β-hydroxy-Δ^5^-steroid) from cholesterol via a series of three sequential chemical mono-oxygenation reactions. Initially, two hydroxylations that form 22β-hydroxycholesterol and 20α,22β-dihydroxycholesterol followed by a final lyase reaction that results in cleavage of the cholesterol side chain C20-22 bond [34]. It is the very presence of this enzyme, as seen in studies in the CNS below, that in essence determines whether a cell is ‘steroidogenic’ [25]. At this stage pregnenolone is oxidised into progesterone (a 3-oxo-Δ^4^-steroid) in an NAD^+^-dependent manner by the inner mitochondrial membrane enzyme 3β-hydroxysteroid dehydrogenase (3β-HSD) [35,36], acting within the acidic mitochondrial intermembrane space [37]. This enzyme is expressed in CNS and peripheral tissues and also catalyses the conversion of DHEA (also a 3β-hydroxy-Δ^5^-steroid) into androstenedione (like progesterone a Δ^4^-steroid) [38]. Pregnenolone can also be converted into 17-hydroxypregnenolone by the cytochrome P450 17A1/17α-hydroxylase (P450c17), which can, in turn, be converted to 17-hydroxyprogesterone by 3β-HSD [39]. Progesterone passes into the cytosol via passive diffusion across the outer mitochondrial membrane [25] and can then be further metabolised to 5α-dihydroprogesterone by steroid 5α-reductase, one of the first steroidogenic enzyme activities identified in brain tissue [40], in the ER [41], or to 5β-dihydroprogesterone by steroid 5β-reductase [42]. These steroids are then converted to 3α,5α-tetrahydroprogesterone (commonly referred to as allopregnanolone) by 3α-hydroxysteroid dehydrogenase (3α-HSD) [9] or 3β,5α-tetrahydroprogesterone (referred to as pregnanolone) by 3β-HSD [43]. The 5α-reductase enzyme and 3α-HSD operate as a complex that is expressed widely across the brain [44].

The P450c17 enzyme can also convert progesterone into 17-hydroxyprogesterone, which, along with 17-hydroxypregnenolone, can be further converted by P450c17 into androstenedione and DHEA, respectively [45]. The enzyme 17β-hydroxysteroid dehydrogenase (17β-HSD) then converts DHEA and androstenedione into androstenediol and testosterone respectively [46]. The 3β-HSD enzyme can also convert DHEA and androstenediol into androstenedione and testosterone [47]. The cytochrome P450 aromatase enzyme (P450aro) then converts androstenedione and testosterone into estrone and estradiol respectively [48]. Finally, estrone can be converted into estradiol by 17β-HSD [45], whilst testosterone can be converted by 5α-reductase into 5α-dihydrotestosterone (DHT) and then by 3α-HSD into androstanediol [49]. Although not discussed in this review, for information on corticosteroids (Figure 2) and their role in the brain there are recent detailed commentaries [50,51].

#### Identification of neurons and glia of the CNS and PNS as sites of neurosteroid synthesis and function

At the tissue level, various brain areas have been shown to express StAR, TSPO and the P450scc enzyme [52–55], which exert strong control over the production of pregnenolone. Furthermore, the mRNA of 3β-HSD, responsible for progesterone production, has been detected in various brain areas [23,53,56]. In rat brain sections, glia were first considered as the primary site of neurosteroid synthesis owing to the presence, by immunocytochemistry, of P450scc in the white matter [57]. Furthermore, TSPO immunoreactivity has also been demonstrated in brain white matter [58]. TSPO expression was shown to increase in response to injury, suggesting a role of neurosteroids in brain repair [59,60]. The earlier discoveries of P450scc immunohistochemistry were subsequently supported by RT-PCR [61], with both data sets confirming biochemical observations that mitochondria from the myelin producing oligodendrocytes could convert 3H-mevalonate to pregnenolone [62]. Since these initial studies, the presence of 3β-HSD, 5α-reductase and 3α-HSD have been shown in mixed cultures of oligodendrocytes and astrocytes, pure type I astrocyte cultures, and regions of the brain corresponding to glia and oligodendrocytes [63–68]. These three enzymes sequentially convert pregnenolone into progesterone, 5α-dihydroprogesterone and finally allopregnanolone respectively (Figure 2). Glia are also the only confirmed site (*in vitro*) of DHEA synthesis from pregnenolone [69]. The ability of glial cells to generate neurosteroids may be related to their requirement during differentiation. Gago and colleagues reported in 2001 that the activity of 5α-reductase is 5 times higher in mature oligodendrocytes compared with glial progenitors leading to increased formation of the allopregnanolone precursor 5α-dihydroprogesterone [70]. The production of neurosteroids is not restricted to oligodendrocytes of the CNS. Myelinating PNS Schwann cells also express the relevant enzymes and synthesize neurosteroids (reviewed in [5]) as evidenced by the ∼10 times higher levels of progesterone inside these cells than are found in plasma [71]. All myelinating cells are therefore capable of steroidogenesis, which is itself a critical metabolic component of the process of myelination [71]. Indeed, this was evidenced by a seminal study from 1995 using a mouse PNS axon lesion model in which it was shown that levels of pregnenolone and progesterone remain elevated during the process of remyelination [71]. Furthermore, disruption of progesterone biosynthesis or function by small molecule inhibitors results in defective remyelination [71]. The specific roles of neurosteroids in the function of oligodendrocytes and in the process of myelination have been reviewed in more detail elsewhere [72].

Despite a large body of work on the role of glia in neurosteroid biosynthesis, considerably less was initially known about this process in neurons. Studies by Tsutsui and the group of Baulieu were the first to demonstrate the presence of neuronal neurosteroid biosynthesis machinery in the brain. First came the identification of P450scc in cerebellar Purkinje neurons by immunohistochemistry in quail brain [73]. This was followed almost immediately by the identification of 3β-HSD by *in situ* hybridization in neurons throughout the rat brain including in the hippocampus, cortex and cerebellum, with strongest expression confirmed by immunohistochemistry to neurons of the cerebellum [74]. Confirmation of the important role of cerebellar Purkinje neurons in brain neurosteroid production came from further immunostaining and RT-PCR studies demonstrating P450scc expression [55]. Cerebellar Purkinje neurons have one of the most recognised architectures of any neuron with extensively branched dendrites and large cell bodies forming the characteristic Purkinje cell layer that resides between the molecular and the granule cell layers. Purkinje neurons are therefore well equipped and well placed to receive thousands of excitatory inputs from parallel fibres and single climbing fibres of the cerebellum [75]. Remarkably, Purkinje cells, which are GABAergic and therefore inhibit neurotransmission elsewhere in the CNS, are the only neuron that carries outputs from the cerebellum to the cortex [75]. Although traditionally associated with motor and balance function, the cerebellum also has roles in cognitive and affective behaviour [76]. Subsequently, neurons from various parts of the brain including the hippocampus, dentate gyrus and cerebellar granule layer, as well as Purkinje neurons, have been demonstrated to contain the necessary machinery for *de novo* synthesis of neurosteroids including; pregnenolone, pregnenolone sulfate, progesterone and progesterone metabolites (allopregnanolone) [52]. The expression of StAR, P450scc, 3β-HSD and 5α-reductase have been measured in neurons from differentiation through development and into adulthood [52,55,56]. It is known that the requirement for neurosteroids in postnatal development is substantial, with high levels of progesterone and metabolites measured biochemically [55,56,77,78] alongside increasing expression of 3β-HSD to produce progesterone [56]. Interestingly, neurons have been shown to contain higher amounts of 5α-reductase activity compared to glia whilst type 1 astrocytes express more 3α-HSD [66]. As described above, these enzymes form a complex to regulate the production of allopregnanolone, which is believed to be essential for the survival of Purkinje and granule neurons during cerebellar development [79,80]. This observation of differential expression of neurosteroidogenic genes in different cells of the CNS may provide a mechanism for the generation and release of neurosteroids in order to mediate their extracellular signalling effects [81], which are discussed in more detail below. Support for a specific role of neurosteroids in the organisation and development of cerebellar circuitry came from studies showing that progesterone could promote dendritogenesis in Purkinje cells *in vitro* in rat cerebellar slice cultures, and promote Purkinje spine density *in vivo* [77,78]. At the whole organism level, it has been shown that treatment with pregnenolone, and several other neurosteroids, including DHEA and their sulphated derivatives, enhanced task-associated learning during footshock avoidance tests in mice [82]. This is supported by the findings that infusion of pregnenolone sulfate into the nucleus basalis magnocellularis enhanced task associated memory in rats, whilst an infusion of tetrahydroprogesterone had the opposite effect [83]. These effects were explained by the impact of these neurosteroids on GABA_A_ receptor (GABA_A_R) signalling (detailed below), with pregnenolone sulfate being a GABA_A_R antagonist and tetrahydroprogesterone being a GABA_A_R agonist at the concentrations used in the study.

Beyond neurons and glia, there is also evidence that the pineal gland is able to synthesise neurosteroids. The pineal gland is a small endocrine organ located in the epithalamus, between the cerebellum and the cortex, and is involved in the production of melatonin for the modulation of circadian and seasonal cycles [84]. The pineal gland has been shown to secrete neurosteroids in response to light in chicks, and during quail cerebellar development, potentially promoting Purkinje cell survival by suppressing apoptosis [80,85]. These observations are further supported by the findings that removal of the pineal gland leads to loss of Purkinje neurons during chick development [86]. However, despite the complementary *in vitro* and *in vivo* data, it is still unclear as to whether the CNS functions of neurosteroids are derived solely from synthesis within the CNS or whether there is a contribution of adrenal gland/gonad steroids.

#### Modulators of neurosteroid biosynthesis

Very little is currently known about whether neurosteroidogenesis is triggered or regulated by extracellular or other physiological stimuli. With respect to intracellular second messengers, rat glia and C6 glioma cells have been shown to respond to elevations in cyclic adenosine monophosphate (cAMP) and subsequent activation of protein kinase A (PKA) mediated signalling pathways by rapidly increasing the transcription of 5α-reductase mRNA which in turn increases enzyme levels and activity [87,88]. In addition to elevation of 5α-reductase, StAR and TPSO have both been shown to play a role in neurosteroidogenesis linked to the activation of glial cells [89]. Both are phosphorylated by PKA leading to increased influx of cholesterol into mitochondria via increased activity of StAR and alteration of the ligand binding affinity of TPSO [90]. The neurosteroid estradiol has also been shown, in female rat hypothalamic astrocytes, to bind to the metabotropic glutamate receptor 1 (mGluR1) and induce Ca^2+^ release mediated by inositol 1,4,5-trisphosphate [91], subsequent activation of Ca^2+^ dependent adenylyl cyclase, which in turn elevates cAMP and triggers PKA-mediated phosphorylation of TSPO and StAR [92]. One potential function of this signalling cascade is regulation of the luteinizing hormone surge, which triggers ovulation [93]. Circulating estradiol of the developing follicles triggers synthesis of progesterone, by elevating 3β-HSD expression, in astrocytes of the female hypothalamus [91]. This in turn induces changes in gonadotropin neuron synaptic plasticity and enhanced secretion of luteinizing hormone [93], which can be blocked by inhibition of progesterone biosynthesis [91]. This example is therefore one demonstration of how peripheral steroid hormones integrate with neurosteroidogenesis and neurosteroid signalling.

### Signalling functions of neurosteroids

Predating the knowledge that neurosteroids were synthesized within the brain, reports of brain targeted function of steroids has existed for some time. Steroid hormones have been shown to produce anti-seizure like properties, anticonvulsant, sedative and anaesthetic effects [94–96]. Acute effects of neurosteroids on the brain are typically not mediated by classical steroid hormone mechanisms as neurosteroids are not active upon these intracellular sites; instead they mediate their effects on neuronal excitability via ion channels and neurotransmitter receptors, primarily GABA_A_ receptors [97]. Here, we will review the opposing effects of neurosteroids on GABA_A_ and NMDA receptors [97–99]. Neurosteroids also act on several other ion channel receptors, including glycine, AMPA and sigma receptors, which have recently been reviewed by Rebas et al [100].

#### GABA

GABA_A_ receptors (GABA_A_R) are the major mediators of inhibitory neurotransmission in the CNS. They are heteropentamers, with abundant receptors comprising α, β and γ or δ subunits. There are a wide array of different GABA_A_R configurations, which confer different electrophysiological and pharmacological properties, reviewed here [101]. The activity of GABA_A_R is modulated by several classes of molecule, including neurosteroids, benzodiazepines, and barbiturates, which reduce neuronal excitability and mediate anxiolytic and sedative effects. Neurosteroids can be positive or negative modulators of GABA_A_R activity. Potentiating neurosteroids, such as allopregnanolone, tetrahydrodeoxycorticosterone (THDOC) and androstanediol, are positive modulators of GABA_A_R activity with two distinct modes of action. At high concentrations (>100 nM), neurosteroids can directly activate GABA_A_R [102,103]. However, neurosteroids are also potent positive modulators of GABA_A_R activity, acting via two different mechanisms depending on ambient GABA concentration. Low nanomolar neurosteroid concentrations extend the open time of GABA_A_R exposed to a low concentration of GABA for a prolonged period, and thus enhance inhibitory neurotransmission [104]. This is physiologically relevant to extrasynaptic GABA_A_R, which experience these conditions. In studies that mimic the short exposure to saturating levels of GABA relevant to synaptic GABA_A_R, neurosteroids extend the delay of GABA_A_R mediated current, with a delay to receptor recycling a proposed mechanism [105]. Inhibitory neurosteroids, which mostly comprise sulphated neurosteroids such as pregnenalone sulfate and DHEA sulfate, act as non-competitive GABA_A_R antagonists [99,106,107].

Neurosteroids can influence a wide range of GABA_A_R subtypes; this is supported by the identification of the proposed potentiating neurosteroid-binding site close to the membrane between subunits [108]. A homopentameric α subunit transmembrane domain construct has been crystallised with the potentiating neurosteroid THDOC, which revealed binding between the subunits. This binding site has been modelled in the most abundant α1β2γ2 arrangement, revealing that binding pockets capable of accommodating neurosteroids in the correct binding orientation are found between all the subunits [109]. This is different to a previously proposed neurosteroid binding site in the transmembrane domains of the α subunit [110], but is the only site supported by structural data. The promiscuity of neurosteroids with regard to GABA_A_R subtypes is therefore not surprising. There are, however, differences in the extent to which different GABA_A_R subtypes are modulated. At low ambient GABA levels, such as those typically experienced by extrasynaptic GABA_A_R, neurosteroids mediate their potentiating effects mostly via GABA_A_R that contain a δ subunit; these effects are almost abolished in neurons from mice lacking the δ subunit [111–113]. This difference is not thought to be due to neurosteroid binding by the δ subunit, but is rather to do with the mechanism of activation of δ containing GABA_A_R by GABA [110]. GABA_A_R containing δ subunits are located extrasynaptically, where they mediate tonic inhibitory currents. Although GABA binds to these receptors with high affinity, it only acts as a partial agonist. It is thought that neurosteroids increase the likelihood of channel opening following GABA binding, thus converting GABA to a full agonist [114]. As GABA already acts as a full agonist at γ containing GABA_A_R, there is less scope for this type of potentiation.

The site of inhibitory neurosteroid binding to GABA_A_R is unknown. However, it is thought to be outside the pore, and distinct from the binding sites of both potentiating neurosteroids and picrotoxinin (GABA_A_R open channel blocker) binding [115]. The evidence for this is that inhibition is not voltage-dependent, as would be expected for an open channel blocker, the inhibitory effects of pregnenolone sulfate and picrotoxin are additive, and, unlike potentiating neurosteroids, pregnenolone sulfate on the inner membrane leaflet cannot affect GABA_A_R. Neurosteroid mediated GABA_A_R inhibition is more effective at higher GABA concentrations, suggesting that the binding site is exposed by receptor activation [116]. This is supported by the greater inhibition of steady state, rather than peak, current through GABA_A_R. Similarly to potentiating neurosteroids, inhibitory neurosteroids act across a wide range of GABA_A_R configurations, but, unlike potentiating neurosteroids, are more effective at GABA_A_R that contain γ subunits [117,118].

#### NMDA

NMDA receptors (NMDAR) are a class of ionotropic glutamate receptor, found pre-synaptically, post-synaptically and extra-synaptically, that mediate excitatory neurotransmission. They take the form of heterotetramers, made up of a dimer of dimers, with each dimer comprised of a GluN1 and GluN2 or GluN3 subunit. Variation within each subunit class further increases NMDA receptor diversity. Unlike other glutamate receptors, NMDA receptors require co-stimulation with glycine or D-serine for activation [119]. As with GABA_A_R, neurosteroids can both positively and negatively modulate receptor activity. The sulphated neurosteroid pregnanolone sulfate is a non-competitive NMDAR inhibitor, with the greatest inhibitory effects on receptors containing the GluN2C and GluN2D subunits [120,121]. This inhibitory effect is achieved via decreasing channel open probability and open time, and by increasing receptor desensitisation [122].

In contrast to GABA_A_R, where sulphated neurosteroids act solely as inhibitors, pregnenolone sulfate has pleiotropic effects on NMDAR. Classically, it has been confirmed numerous times to potentiate NMDAR activity, including [98,120,123–126]. This potentiation is subtype-dependent, acting only on receptors that contain the GluN2A or GluN2B subunits, with the greatest effect on GluN2A-containing receptors [98,120,126]. Pregnenolone sulfate potentiates NMDA receptors by increasing channel open probability and slowing receptor deactivation [98,126]. The greater effect on GluN2A containing receptors is due to their faster native deactivation rate; the rate for GluN2A and GluN2B containing receptors is similar in the presence of pregnenolone sulfate [98]. Also in contrast to the situation with GABA_A_R, the potentiating effect of pregnenolone sulfate on NMDAR is dependent on binding to inactive receptors. The potentiating effect of pregnenolone sulfate is much greater when cells are pre-treated than when it is co-applied with glutamate, suggesting that it cannot bind to activated receptors [127]. In addition to this potentiation, pregnenolone sulfate can directly activate GluN1/2A and GluN1/2B NMDAR [128], making its actions on these NMDAR analogous to the actions of potentiating neurosteroid modulation of GABA_A_R. The final modulation of NMDAR by pregnenolone sulfate is as an inhibitor; it inhibits NMDAR that contain GluN2C and GluN2D subunits [98,120,127].

The mechanistic basis of neurosteroid modulation of NMDAR is unclear. Pregnenolone sulfate and pregnanolone sulfate bind at different sites, and exert their effect from the outer leaflet of the plasma membrane, suggesting that the site of synthesis and site of action are distinct [124]. More specifically, the M3-M4 extracellular loop of GluN2 subunits has been found to be vital for neurosteroid modulation of NMDAR [126,129]. Pregnanolone sulfate inhibition of NMDAR is voltage-independent, suggesting that it binds outside the pore region, but is usage-dependent, with binding only seen in the presence of receptor agonist [129]. Modelling studies suggest that pregnanolone sulfate binds extracellularly to resting receptors, and that agonist binding causes a conformational change, allowing pregnanolone sulfate to move into the pore region and block ion conductance [130]. More is known about pregnenolone sulfate binding. A steroid modulatory domain has been identified in the GluN2B subunit, comprising parts of the glutamate binding site, and the M4 transmembrane region. Models indicate that residues in this domain contribute to a hydrophobic binding pocket between subunits that could accommodate pregnenolone sulfate [131]. More recently, pregnenolone sulfate and pregnanolone sulfate have been shown to bind different sites in the extracellular amino terminus of GluN2D, and to the S1/S2 domain of GluN2B [132]. However, as these experiments used soluble fragments and saturating neurosteroid concentrations, the physiological relevance of this binding is uncertain.

### Impact of neurosteroids in neurodegenerative disease

#### Diseases of steroid biosynthesis and transport

There are surprisingly few studies on how disrupted sterol biosynthesis or sterol transport associated to human diseases impact upon neurosteroid levels and their signalling functions. Sterols are either created *de novo* within cells via the action of 3-hydroxy-3-methyl-glutaryl coenzyme A (HMG CoA) reductase to generate mevalonate or are brought into the cell via LDL receptors [133]. With respect to the LDL receptor pathway, apolipoprotein E (ApoE), which binds with high affinity to the LDL receptor, is associated with Alzheimer disease (AD) and altered neurosteroid levels (described in detail below). At the lysosome, LDL receptors release their cargo and free cholesterol is liberated by the action of acid lipase [133]. To our knowledge, no studies have been reported determining whether neurosteroids are altered in lysosomal acid lipase deficiency, a lysosomal storage disease with accumulation of cholesteryl esters and triglycerides in cells and tissues [134]. Once cholesterol is released, it is mobilised within the lumen of lysosomes by the NPC2 protein, which delivers the cholesterol to the lysosomal transmembrane protein NPC1 [135]. This protein, or others such as LIMP2, may transfer the cholesterol out of lysosomes via ER-lysosome contact points [136]. Loss-of-function mutations in the genes that encode either NPC1 or NPC2 result in the neurodegenerative lysosomal multi-lipid storage disease Niemann-Pick type C (NPC) [135]. Lysosomal cholesterol storage in NPC disease was found to associate with age-dependent reductions in the activity of 3α-HSD [79] and the biochemical levels of estradiol [92] in the brains of the NPC1 protein deficient *Npc1^nih^* mouse model. The deficiency in estradiol secretion by the *Npc1^−/−^* mouse glia was shown in co-culture experiments to be less supportive of the growth of wild-type neurons, with fewer and shorter neurites measured [92]. A more substantial role for neurosteroids in the pathogenesis of NPC disease was suggested in Griffin et al. whereby treatment with allopregnanolone, solubilised in cyclodextrin, could compensate for functional absence of the synthesis of this neurosteroid. With either a single post-natal day 7 injection [79], or in a subsequent study by multiple injections [137], allopregnanolone solubilised in a 20% (w/v) solution of 2-hydroxypropyl-β-cyclodextrin (HPβCD) led to a dramatic increase in the life expectancy of the mouse model from 67 days to 124 days by single injection or even longer by multiple injections. However, subsequent studies by the groups of Dietschy and then Walkley identified that this effect was mostly mediated by the cyclodextrin carrier alone, with only a small additive effect when both HPβCD and allopregnanolone were tested together versus the HPβCD vehicle alone [138,139]. These findings, alongside many others, ultimately led to a clinical trial of HPβCD in NPC patients [140,141]. Furthermore, allopregnanolone administered in complex with other solubilising agents such as corn oil had no therapeutic effect on disease phenotypes or life expectancy [139], suggesting that the efficacious molecule in all NPC disease studies whereby allopregnanolone is combined with HPβCD is, in fact, the HPβCD carrier. In the case of estradiol, a subcutaneous injection of 2 or 20 mg/kg estradiol solubilised in corn oil at post-natal day 7 leads to a modest improvement in life expectancy alongside some functional improvement on the rota-rod test [142]. It therefore appears, for now, that the impact of deficient neurosteroid biosynthesis on NPC disease pathogenesis is relatively minimal [79,143]. Beyond the endosomal system, cholesterol is transported to lysosomes by StARD proteins, such as StARD3, and then into the inner mitochondrial membrane as outlined above. Diseases associated with StAR and P450scc have been recently reviewed in detail elsewhere [143], whilst diseases of the steroid hormone biosynthesis pathway, including mutations in the *CYP7B1* gene, encoding 25-hydroxycholesterol 7α-hydroxylase, which cause hereditary spastic paraplegia [144], have also been reviewed [145].

#### Multiple sclerosis, neurosteroids and myelination

Multiple sclerosis (MS) is a grey and white matter demyelinating disease of the CNS, characterised by episodes of relapse and remission. The disease is common, with an estimated incidence of 1/320 in the U.S.A. [146]. At the cellular level, MS presents with oligodendrocyte dysfunction and loss, demyelination and axon injury, neurodegeneration and subsequent neuroinflammation [147]. Steroid hormones have been implicated in MS as a result of the findings that (1) disease relapse is less likely to occur during the final 3 months of pregnancy and more likely to relapse during the first 3 months after birth, which fits with elevated and then reduced levels of oestrogen and progesterone respectively [148] and (2) incidence and progression are sex-dependent [149]. In addition, the brains of MS patients and the MS mouse model (experimental autoimmune encephalomyelitis) present with reduced allopregnanolone and DHEA levels, alongside reduced expression of neurosteroid biosynthetic enzymes [147,150]. Furthermore, treating MS model mice with progesterone led to a correction in neurosteroid production [151,152], reduced neuroinflammation and reduced demyelination [151,153]. Although multiple therapeutic options exist for MS, neurosteroids appear to be another promising avenue for treating this disease.

#### Alzheimer disease

An estimated ∼47 million people worldwide are living with dementia, of which Alzheimer disease (AD) is the most common cause (60–80%) [154]. Classically, the disease is characterised by extracellular plaques composed of amyloid beta (β-amyloid) peptide and intracellular neurofibrillary tangles of the tau protein [154]. In addition to rare genetic forms of AD, the only gene initially associated to increased risk of AD was *APOE4*, encoding an apolipoprotein lipid carrier associated with cholesterol transport [154]. More recently, GWAS studies have identified components of the sterol pathway as increasing the risk of developing AD, all of which point to a role for sterol homeostasis in AD pathophysiology [155]. With respect to neurosteroids, lower levels of allopregnanolone (and other neurosteroids) have been shown in AD patient plasma and serum [156,157]. Furthermore, lower levels of allopregnanolone have also been shown in post-mortem samples from the prefrontal and temporal cortex of male and female AD patients compared with controls. These changes were inversely correlated to the Braak disease stage [158,159] and positively correlated with the *APOE4* AD risk gene [159]. In contrast to the lower levels of allopregnanolone, higher levels of pregnenolone and DHEA have been measured in both cerebrospinal fluid and postmortem brain tissue of AD patients, which correlate with the Braak stage of pathology [158–162]. Both pregnenolone and DHEA have been shown to provide a protective effect against cellular [163–165] and *in vivo* [166] toxicity induced by the β-amyloid protein, suggesting a protective effect. However, as levels of DHEA have been shown to decrease naturally with age [167], and as β-amyloid addition to cells triggers an increase in levels of DHEA [161], it is currently unclear whether the increase in DHEA in AD samples is a compensatory mechanism or a direct component of the disease. As the sterol pathway has been implicated in the risk of AD [155] and as the β-amyloid protein can bind to sterols [168], it is tempting to speculate that neurosteroids may be directly implicated in AD pathogenesis.

#### Neurosteroids as therapeutic molecules and biomarkers

**Figure 3:**
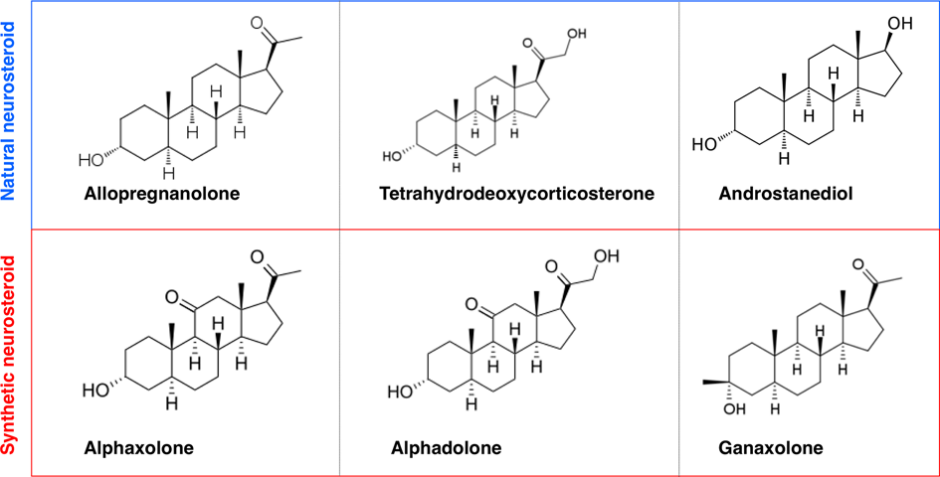
Synthetic neurosteroids used in therapeutic studies and their natural neurosteroid analogue

Multiple synthetic analogues of neurosteroids exist (Figure 3), some of which have previously been in widespread therapeutic use since the 1970s, namely alphaxalone and alphadolone [169] dissolved in 20% solution of polyoxyethylated castor oil surfactant [170]. A combination of two neurosteroids were used as alphadolone was less potent than, but increased the solubility of, alphaxolone, which is a powerful but short acting anaesthetic with minimal side effects [170]. However, this synthetic neurosteroid mixture was removed from human clinical use in the 1980s owing to adverse anaphylactic effects incurred by the solubilising agent. However, it remains in veterinary use under the trade name of Saffan [169]. More recently, the synthetic neurosteroid ganaxolone, a GABA_A_R modulator, has been in clinical trials for epilepsy, as it protects against seizures in animal models, with some evidence of efficacy in humans [171]. Several other neurosteroid analogues have been in clinical trials (e.g. for Fragile X syndrome and autism), although with mixed outcomes, reviewed here [169,172–174]. The implications of the use of various neurosteroids as therapeutic molecules in several human diseases and medical conditions have been described in detail elsewhere [169] and are therefore not covered in any further detail here. In conjunction with the development of neurosteroids as therapeutics, their use as disease biomarkers has also been suggested. This avenue of research appears to be supported by the findings that cerebrospinal fluid levels of pregnenolone and DHEA correlate with concentrations within the temporal cortex and that both are elevated in AD patient cohorts [160]. Further evidence from rodent experiments indicates that changes in serum pregnenolone levels correlate sufficiently with concentrations in the hippocampus [175]. However, plasma levels of DHEA and its sulphated form have been reported to be lower in AD patients [176]. Furthermore, any measurements may be clouded by the transfer of peripheral blood steroids into the brain [177], alongside the aforementioned potential for errors in measurement of some sulphated neurosteroids [8].

## Conclusions and perspectives

Despite significant progress in our understanding of neurosteroids over the last 30+ years, much work remains to fully elucidate their roles in maintaining healthy function of the CNS and PNS. There is evident therapeutic potential for neurosteroids, as exemplified by the recent approval of brexanolone, an allopregnanolone formulation, for postpartum depression [178]. However, this potential remains largely under-developed. Difficulties associated with their nomenclature, measurement, reported effects and development as therapeutic molecules may be hindering the expansion, and evident potential, of this field.

## Summary

- Neurosteroids are generated *de novo* in peripheral and central nervous system tissues.
- Neurosteroids, in contrast to peripherally generated adrenal steroid hormones, directly target extracellular receptors rather than mediate their effects via classical intracellular steroid hormone nuclear receptors.
- Neurosteroids regulate developmental, growth and signalling functions of the CNS and PNS.
- Neurosteroids have emerging roles in neurological disease and have potential to be utilised therapeutically.

## Open Access

Open access for this article was enabled by the participation of Cardiff University in an all-inclusive *Read & Publish* pilot with Portland Press and the Biochemical Society under a transformative agreement with JISC.

## Abbreviations

3α-HSD: 3α-hydroxysteroid dehydrogenase
3β-HSD: 3β-hydroxysteroid dehydrogenase
17β-HSD: 17β-hydroxysteroid dehydrogenase
AD: Alzheimer disease
AllotetrahydroDOC: allotetrahydrodeoxycorticosterone
ApoE: apolipoprotein E
cAMP: cyclic adenosine monophosphate
CNS: central nervous system
CRAC: cholesterol recognition/interaction amino acid consensus
DHEA: dehydroepiandrosterone
DHEAS: dehydroepiandrosterone sulphate
DHT: 5α-dihydrotestosterone
ER: endoplasmic reticulum
GABA: γ-aminobutyric acid
GABA_A_R: γ-aminobutyric acid receptors, type A
GWAS: genome wide association study
HMG CoA: 3-hydroxy-3-methyl-glutaryl coenzyme A
HPβCD: 2-hydroxypropyl-β-cyclodextrin
LDL: low density lipoprotein
LIMP: lysosomal integrated membrane protein
mGluR1: metabotropic glutamate receptor 1
MS: multiple sclerosis
NMDA: N-methyl-D-aspartic acid
NMDAR: NMDA receptor
NPC: Niemann-Pick type C
P450aro: cytochrome P450 aromatase
P450c11β: sterol 11β-hydroxylase
P450c17: cytochrome P450 17A1/17α-hydroxylase
P450c21: steroid 21-hydroxylase
P450scc: cytochrome P450 side chain cleavage
PKA: protein kinase A
PNS: peripheral nervous system
PREGS: pregnenolone sulphate
RT-PCR: reverse transcriptase polymerase chain reaction
StAR: steroidogenic acute regulatory protein
StARD: steroidogenic acute regulatory domain
THDOC/TetrahydroDOC: tetrahydrodeoxycorticosterone
TSPO: translocator protein
